# New insight into Epstein-Barr virus infection using models of stratified epithelium

**DOI:** 10.1371/journal.ppat.1011040

**Published:** 2023-01-11

**Authors:** Ian R. Hayman, Rachel M. Temple, Cole K. Burgess, Mary Ferguson, Jason Liao, Craig Meyers, Clare E. Sample

**Affiliations:** 1 Department of Microbiology and Immunology, The Pennsylvania State University College of Medicine, Hershey, Pennsylvania, United States of America; 2 Department of Public Health Sciences, The Pennsylvania State University College of Medicine, Hershey, Pennsylvania, United States of America; 3 The Penn State Cancer Institute, Hershey, Pennsylvania, United States of America; University of North Carolina at Chapel Hill, UNITED STATES

## Abstract

Epstein-Barr virus (EBV) is a ubiquitous human pathogen that is transmitted in saliva. EBV transits through the oral epithelium to infect B cells, where it establishes a life-long latent infection. Reinfection of the epithelium is believed to be mediated by virus shed from B cells, but whether a latent reservoir can exist in the epithelia is unknown. We previously developed an *in vitro* organotypic model of stratified epithelium where EBV can readily replicate within the suprabasal layers of the epithelium following apical infection mediated by virus-producing B cells. Given that infected epithelial cells and cell-free virus are observed in saliva, we examined the ability of both of these to mediate infection in organotypic cultures. Epithelial-derived cell-free virus was able to infect organotypic cultures from the apical surface, but showed enhanced infection of B cells. Conversely, B cell-derived virus exhibited enhanced infection of epithelial cells. While EBV has been detected in basal cells in oral hairy leukoplakia, it is unknown whether EBV can be seen in undifferentiated primary keratinocytes in the basal layer. Undifferentiated epithelial cells expressed proposed EBV receptors in monolayer and were susceptible to viral binding and entry. Integrins, and occasionally ephrin A2, were expressed in the basal layer of gingiva and tonsil derived organotypic cultures, but the known B-cell receptors HLAII and CD21 were not detected. Following infection with cell-free virus or virus-producing B cells at either the apical or basolateral surface of preformed organotypic cultures, abundant infection was detected in differentiated suprabasal cells while more limited but readily detectable infection was observed in the undifferentiated basal cells. Together, our data has provided new insight into EBV infection in stratified epithelium.

## Author summary

Epstein-Barr virus (EBV) infects most people worldwide. Infection is often asymptomatic but can result in infectious mononucleosis or cancers of immune or epithelial cell origin. EBV is passed between individuals in saliva, infects oral epithelium and produces more virus that must pass through the multilayered epithelial barrier to establish a lifelong infection in B cells. EBV infection of B cells is well studied, but mechanisms of epithelial infection are not completely understood. We previously developed an *in vitro* model of EBV infection in multilayered epithelium, where EBV produces new virus in the upper differentiated epithelial layers, which has been observed in patient samples of EBV-infected oral epithelium. Using this model, we found that cell-free EBV can infect multilayered epithelium from either the upper or lower surface, modeling infection from virus in saliva or virus within the body, respectively. EBV was also able to enter cells in the less differentiated layers, consistent with observations from human tissue, suggesting that an epithelial reservoir might be established in oral epithelium. This model can be used to identify viral proteins that mediate infection, which may be additional targets for vaccination, and to further delineate the lifecycle of EBV in the epithelium.

## Introduction

Epstein-Barr virus (EBV) is a ubiquitous human herpesvirus that infects the majority of the population worldwide [1,2]. While most infections are mild or asymptomatic, EBV infection can be associated with numerous malignancies, e.g., Burkitt lymphoma, Hodgkins lymphoma, diffuse large B cell lymphoma, and nasopharyngeal and gastric carcinoma [1,2]. While these cancers are characterized by a maintenance of EBV latency, productive replication is observed in oropharyngeal cells in saliva from infectious mononucleosis patients as well as in the suprabasal layers of oral hairy leukoplakia, epithelial lesions that can occur in the oral cavity of AIDS patients [3]. Suprabasal cells amplify the amounts of EBV produced that can then either reinfect the epithelium or be passed in saliva to a new host, where it can infect epithelium and B cells, ultimately resulting in long-term latency in memory B cells [4–8]. Our knowledge of EBV infection in B cells is extensive, largely due to EBV’s ability to infect and immortalize primary B cells in vitro, and from studies of B cells in vivo, B cell tumors and B cell lines that express specific programs of EBV latency-associated genes [9,10]. By contrast, our understanding of the lifecycle of EBV within epithelial cells has significantly lagged behind due to the inability of EBV to readily infect or productively replicate in epithelial cell lines. More recently, we and others have used organotypic cultures to analyze EBV infection in primary or human telomerase-immortalized stratified epithelium, where active virus replication occurs in the suprabasal layers consistent with early studies indicating that EBV replicates in oral epithelium [8,11–15]. This finding was a significant advance at the time that has allowed investigators to explore facets of the virus lifecycle in epithelial cells.

Herpesviruses express glycoproteins gB and gHgL (the latter being a heterodimer) that function as the core fusion machinery. The transfer of EBV between B cells and epithelial cells is facilitated by changes in the glycoprotein composition of the virion [16]. The glycoproteins gp350 and gp42 play an essential role in infection of B cells by binding to CD21 (also known as CR2) and MHCII (also known as HLA class II), respectively [17–19]. During productive replication in B cells, gp42 binds to newly formed HLA class II within the Golgi and is degraded, resulting in virions with a high ratio of gHgL dimeric complexes relative to gHgL-gp42 trimeric complexes [16]. Virions with reduced gHgL-gp42 trimeric complexes are less efficient at infecting B cells [16,17]. By contrast, epithelial cells do not generally express HLA class II, and thus, produce viral particles with elevated levels of gHgL-gp42 trimeric complexes, which more efficiently infect B cells [16,17]. Therefore, virus produced from epithelial cells are preferentially programmed to infect B cells, and vice versa [16]. EBV fusion with an epithelial cell requires gB and the gHgL complex, the latter of which contains a KGD motif that has been shown to mediate binding to integrin proteins [20]. BMRF2 contains a similar motif, RGD, and peptides encompassing this sequence block EBV entry in polarized epithelial monolayers, suggesting that BMRF2 may also play a role in infection through the basal layer [21]. Although EBV in saliva has high levels of gHgL-gp42 trimeric complexes, implying that they are preprogrammed to infect B cells, EBV is transmitted in saliva, suggesting that epithelial-derived virus can infect epithelial cells when passaged to a new host [22]. The first successful infection of monolayer epithelial cells used B cell-associated virus, and it was proposed that attachment of the virus to the B cell sequestered gp350, thereby exposing a second viral glycoprotein (EBV expresses 11 of these during productive replication) that would mediate epithelial infection [23–25]. The virus must cross the dermis to infect B cells and establish a lifelong latent reservoir of the virus with occasional productive replication induced by B cell differentiation. While B cells have limited capacity to cross the basement membrane and migrate through the epithelium, these cells do accumulate in the dermis of *ex vivo* tonsil explants, suggesting that cell-free EBV released by these cells may cross the basement membrane to infect epithelial cells at the basal layer [26–30].

Several cellular proteins have been reported to function as a receptor for EBV in epithelial cells, including neuropilin 1, ephrin A2 and several integrin proteins [21,31–36]. Integrins are bound by proteins containing an RGD or KGD motif and are readily detected in basal layer epithelial cells [37]. There are several ways that EBV could maintain infection of the oral epithelium independent of B cell infection. Discrete sites of productive infection likely exist throughout the oral cavity, releasing cell-free EBV into the saliva [7]. These viral particles can be transmitted to naïve hosts, but likely also re-infect other sites in the oral epithelium. A second possibility is that EBV could form a latent reservoir within stratified epithelium. This latent infection could be similar to the latency established in B cells or be of a more transient nature [3,14,38]. Indeed, EBER transcripts are detected by RT-qPCR analysis of basal cells collected by laser microdissection, suggesting that undifferentiated epithelial cells may support latent infection by EBV [14].

Using organotypic cultures generated from primary keratinocytes, we have previously demonstrated that EBV can infect stratified epithelium when productively replicating B cells are applied to the apical surface [12]. While this was a significant advance in our ability to study EBV infection in epithelium, this model would have wider utility if other aspects of EBV infection could be modeled. We now demonstrate that both cell-free virus and EBV-infected epithelial cells were able to initiate infection of stratified epithelium from the apical surface. Moreover, both cell-free EBV and cell-associated EBV were able to initiate infection from the basolateral surface, resulting in EBV genomes within both the basal and suprabasal layers. Because EBV likely infects epithelium through the basal and apical surfaces in vivo as cell-free virus, these findings will allow us to explore the mechanisms that EBV uses to infect primary stratified epithelium.

## Results

### Origin of cell-free virus impacts infectivity in organotypic cultures

**Fig 1:**
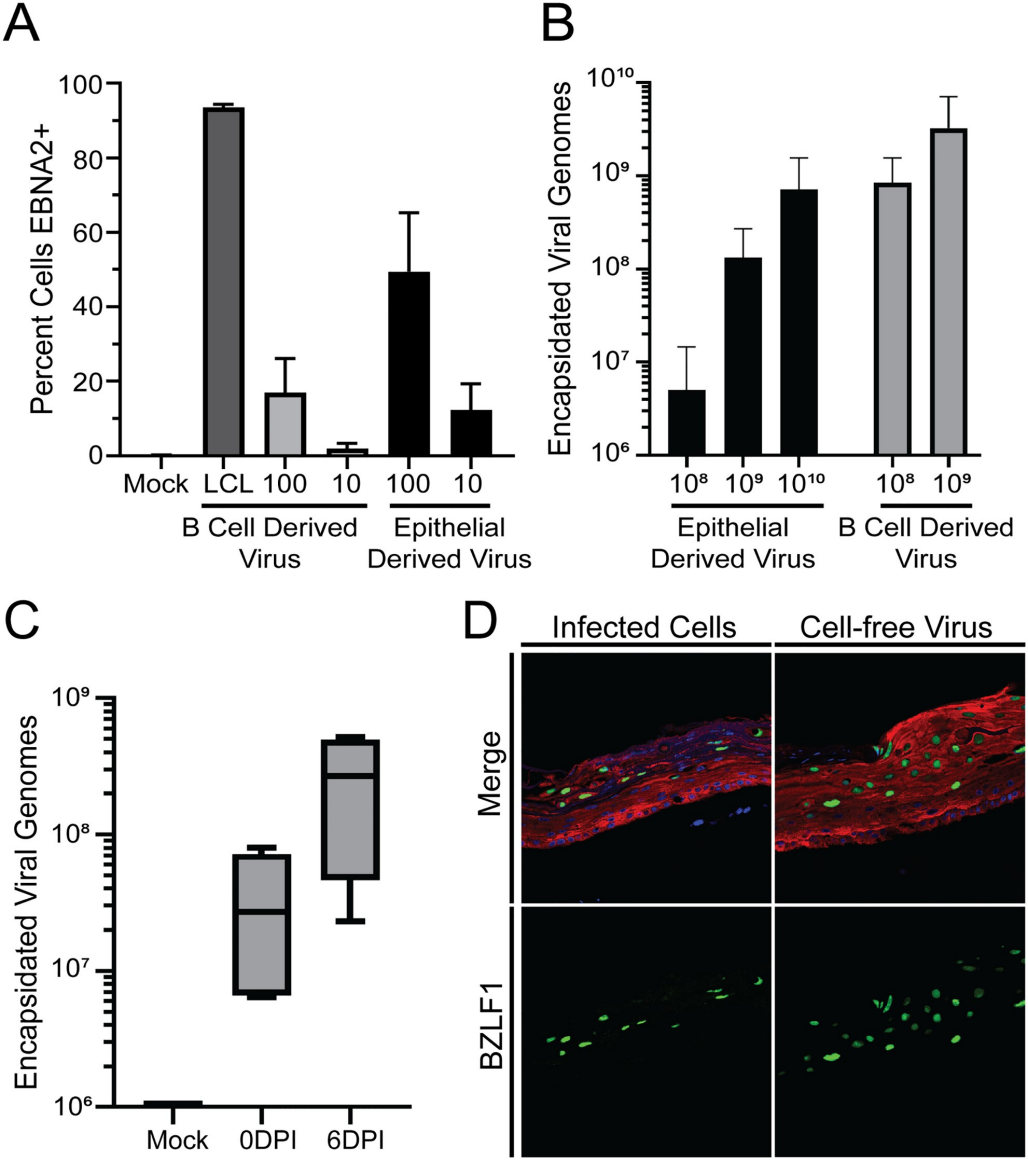
Epithelial-derived virus displays enhanced tropism for primary B cells, while B cell-derived virus has enhanced tropism for epithelial cells. **(A)** Primary B cells were infected with a multiplicity of infection of either 10 MOI or 100 MOI of cell-free virus derived from virus-producing Akata B cells or epithelial organotypic cultures. The number of infected cells was quantified by counting the number of EBNA2-positive cells in approximately 100 cells at 3 days PI. LCLs were used as a positive control for EBNA2 expression. Infections were performed in triplicate. Bars represent the mean ± SD from 2 donor pools of keratinocytes using 1–4 different virus preps for each. Two-way ANOVA analysis determined that infection in primary B cells was significantly impacted by the cellular source of cell-free virus (p < 0.001). **(B)** Scored organotypic cultures were inoculated with cell-free virus containing the indicated number of genomes derived from either Akata B cells or from organotypic cultures and collected 6 days PI. The mean ± SD of encapsidated viral genomes in the organotypic cultures from 3 different donor pools are shown. Infection in organotypic cultures was significantly impacted by the cellular source of cell-free virus as determined by two-way ANOVA (p < 0.005). **(C)** Epithelial cells were isolated from control and infected organotypic cultures at 6 DPI and used to infect a new 4-day organotypic culture. The number of encapsidated genomes present in organotypic cultures 1 hour (0 DPI) and 6 DPI was determined by PCR. **(D)** Expression of the epithelial marker cytokeratin 5 (red) and EBV BZLF1 (green) was assessed in organotypic cultures 6 days PI following inoculation with cells isolated from a separate 6 days PI infected organotypic culture (left) or 1 x 10^8^ epithelial-derived cell-free virus (right). Micrographs are representative images from at least 3 separate donor pools.

While we have previously used B cell-associated virus to infect stratified epithelium from the apical surface, it is not likely that B cell-derived cell-free virus plays an important role in the initial infection of humans [12,16,22]. Previous studies using monolayer epithelial cell lines demonstrated that EBV derived from epithelial cells is better at infecting B cells than is B cell-derived virus, whereas EBV derived from B cells more efficiently infects epithelial cells in monolayer, allowing EBV to efficiently pass between the oral cavity and B cells and vice-versa [16]. To determine whether this preference is maintained in stratified epithelium, we compared the ability of serial dilutions of virus derived from either stratified epithelium or B cells to infect naïve primary B cells and organotypic cultures using equal inoculums of encapsidated virus (Fig 1). For B cell infection, the infected cells were identified by detection of EBNA2 at 3 days post infection (PI). Although virus from both B cells and epithelial cells were able to infect the primary B cells, more EBNA2-positive B cells were observed when infected with epithelial-derived virus (Fig 1A), suggesting that it was more efficient at infecting primary B cells than B-cell derived virus. We then performed the reciprocal experiment in which an equivalent inoculum of virus was used to infect the apical surface of organotypic cultures, and the number of encapsidated genomes detected 6 days PI was used to quantify relative infection. Note that epithelial organotypic cultures support productive EBV replication, but latency-associated proteins have not been detected in organotypic cultures and, thus, EBNA2 is not an appropriate indicator of infection in these epithelial cells. Instead, these experiments assessed virion production as a measure of infection. The results indicated that the B cell derived virus generated considerably more viral progeny in epithelial organotypic cultures than epithelial-derived virus (Fig 1B). Thus, our data indicate that greater infection was seen in the organotypic cultures with B cell derived virus compared to epithelial-derived virus, whereas epithelial-derived virus is more efficient at infecting B cells. These results are consistent with the earlier findings of Hutt-Fletcher, demonstrating that even in the context of the more biologically relevant stratified epithelium, EBV produced from B cells preferentially infects epithelial cells and vice-versa [16].

### Virus-producing epithelial cells can transmit infection between organotypic cultures

EBV in saliva has a glycoprotein profile suggesting that it is derived from epithelial cells [16,22]. We observed that infection with epithelial-derived cell-free virus resulted in poor infection and/or replication and amplification (Fig 1B). Epithelial cells are detected in the saliva, including EBV-infected cells detected during periodontitis [39,40]. Thus, we considered the possibility that organotypic cultures might be efficiently infected by epithelial derived cell-associated virus, similar to what has been reported for B cell associated virus [12,40]. To test this possibility, we harvested cells from infected organotypic cultures and added them to the apical surface of an uninfected organotypic culture that had been grown for 4 days at the air-liquid interface. To quantify the input virus, organotypic cultures were harvested at 1 hour PI, and the amount of virus was quantified as described for Fig 1B. A second set of organotypic cultures were harvested at 6 days PI to evaluate whether EBV had replicated within the epithelium. The number of encapsidated genomes detected in these latter organotypic cultures increased significantly between initial infection and harvest 6 days later (Fig 1C), suggesting that EBV was able to infect the stratified epithelium and initiate productive infection to amplify the virus. The BZLF1 protein, which initiates the productive cycle, was clearly expressed in the suprabasal layers that support productive replication (Fig 1D). These findings suggest that infected epithelial cells seen in saliva can initiate infection in stratified epithelium [39, 40].

### Distribution of EBV receptors in primary epithelial cells in monolayer and organotypic cultures

**Fig 2:**
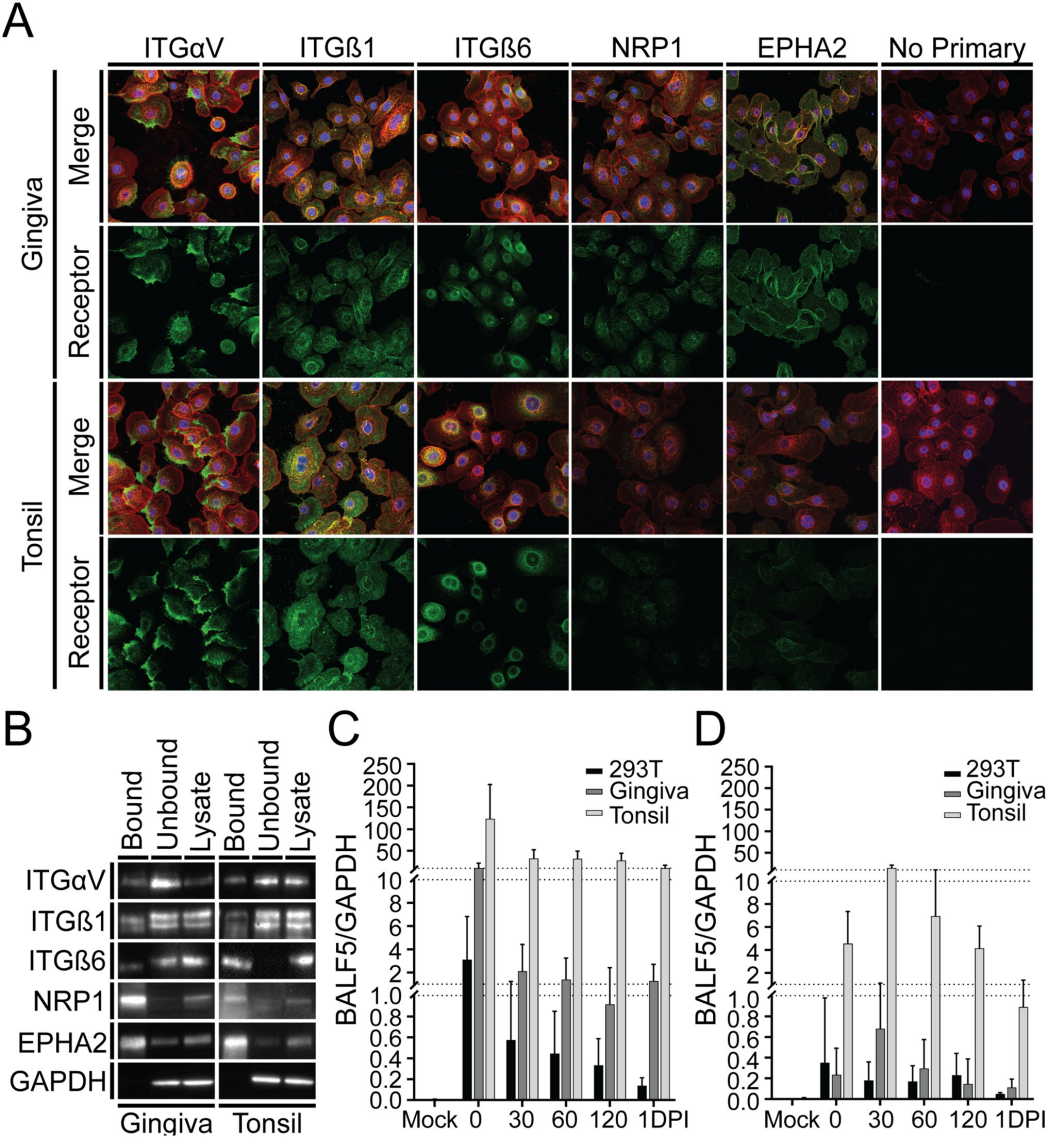
EBV can enter undifferentiated keratinocytes. **(A)** Expression of all proposed epithelial receptors was determined by immunocytochemistry in both gingiva and tonsil cells in monolayer culture (green). Wheat germ agglutinin (red) was used to label the cell membrane, and Hoechst (blue) was used to label cell nuclei. **(B)** Monolayer gingiva and tonsil cells were labeled with biotin, then lysed (lysate). Biotinylated proteins were isolated using streptavidin-labeled paramagnetic particles (bound). EBV was bound to monolayer epithelial cells for 2 hours at 4°C. Excess virus was removed and bound virus allowed to internalize at 37°C for specified times. Cells were treated with proteinase K to remove virus that was not internalized, collected by scraping and pelleted to quantify internalized virus. Total viral genomes in the supernatant fraction containing bound virus **(C)** and the pellet containing internalized virus **(D)** were measured by qPCR and normalized to GAPDH. Bars represent the average ± SD. For all experiments, three biological replicates were performed using two different donor pools.

The cellular proteins that are proposed as EBV receptors in epithelium are integrins alpha V (ITGαV), beta 1 (ITGß1), beta 6 (ITGß6), as well as the non-integrin proteins neuropilin 1 (NRP1) and ephrin A2 (EphA2) based largely on studies in cell lines such as HEK293 cells or EBV-negative cell lines derived from nasopharyngeal carcinoma [21,31–36]. We first evaluated primary gingiva and tonsil keratinocytes grown in monolayers for expression of these proteins. All proteins were detected by immunofluorescence in both gingiva and tonsil keratinocytes, though NRP1 and EphA2 appeared to be expressed at low levels in tonsil cells (Fig 2A). To determine whether these proteins were present at the cell surface, the cells were incubated with biotin to allow labelling of membrane proteins. Proteins were then extracted and incubated with streptavidin-coated beads to isolate the biotin-labelled proteins. Notably, all proteins proposed as EBV receptors were found to be present on the cell surface to varying degrees (Fig 2B). The presence of target candidate receptors, especially integrins, was also readily detected in the unbound fraction. Unbound proteins could either be intracellular or in a location that physically blocks interactions between the protein and biotin label. For example, integrins mediate adhesion of the cell to the growth surface [37]. Thus, integrins in the unbound fraction may represent proteins that are not exposed to the culture media, and consequently viral particles, due to their location between the cell and tissue culture plate.

To assess the ability of EBV to infect primary gingiva and tonsil keratinocytes, the cells were expanded to 80% confluency and incubated with B cell-derived virus at 4°C. The cells were then washed to remove unbound virus, and cells were incubated at 37°C to allow the virus to internalize over a time course up to 24 hours (Fig 2C and 2D). Because HEK293 cells have been used as an epithelial cell line to identify EBV receptors, they were included in this experiment. All cell types bound virus, though with different efficiency, with the greatest binding in tonsil keratinocytes, followed by gingiva and HEK293 cells (Fig 2C). Internalization generally followed the same trend, with tonsil cells appearing to be the most efficient (Fig 2D). These data suggest that cell-free virus can enter undifferentiated epithelial cells, consistent with the expression of multiple epithelial cell receptors for EBV [33,41].

**Fig 3:**
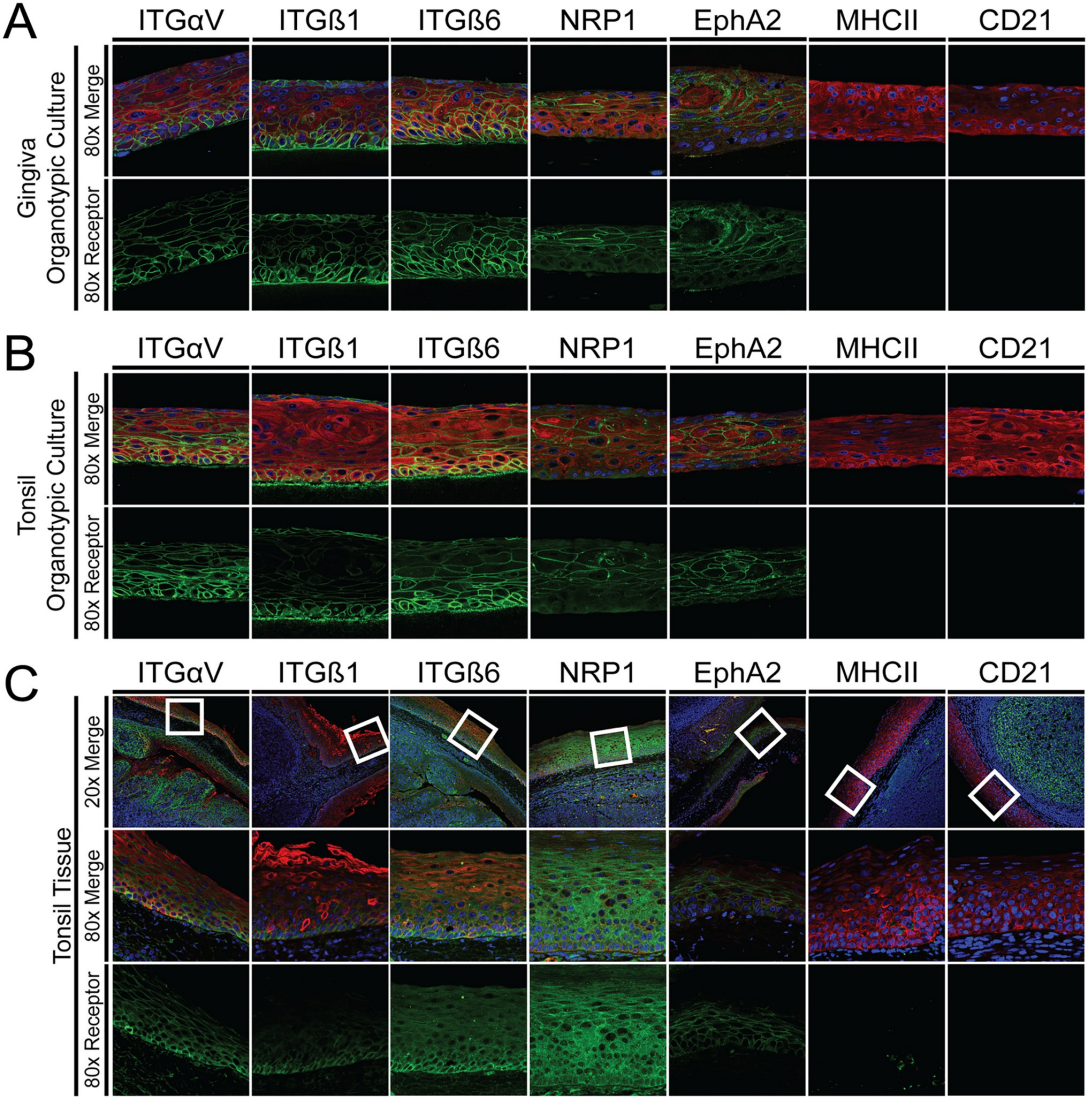
Expression of EBV epithelial cell receptors in gingiva and tonsil organotypic cultures. The expression of proteins proposed to serve as EBV receptors was identified by immunohistochemistry of paraffin embedded sections of gingiva organotypic cultures **(A)**, tonsil organotypic cultures **(B)**, and human tonsil tissue **(C)**. Images are displayed with the basolateral surface at the bottom. Hoechst (blue) was used to detect nuclei, and cytokeratin 5 (red) was used to identify keratinocytes. Proposed epithelial receptors were identified with specific antibodies (green). Micrographs are representative images from three biological replicates from two unique donor pools.

Having shown that primary tonsil and gingiva keratinocytes expressed the EBV epithelial receptors and could be efficiently infected in monolayer culture, we next determined the location and expression of these receptors in stratified epithelium within organotypic cultures. Uninfected gingiva and tonsil organotypic cultures from two separate donor pools were harvested at 4 days after lifting to the air-liquid interface, i.e., the day organotypic cultures are normally infected, and stained for ITGαV, ITGß1, ITGß6, NRP1, EphA2, MHC II, and CD21 (Fig 3). Integrin expression was detected throughout all layers of the organotypic cultures, but was most pronounced in the basal layer. ITGß1 was the most limited in its localization, with clearly greater staining in the lowest layers of the epithelium. There were some patches of cells expressing integrins in the apical layer of the organotypic cultures, which is generally not consistent with integrin expression *in vivo*, and may represent a unique property of integrin expression in this model. The majority of EphA2 was localized to the central layers of the organotypic culture, though there was some limited staining in the lower layers, notably in tonsil organotypic cultures. NRP1 was restricted to the suprabasal layers, with no staining seen in the basal layers of either gingiva or tonsil organotypic cultures. There was no visible staining of MHCII (using an antibody that recognizes HLA-DR/DP/DQ/DX) or CD21 in gingiva or tonsil organotypic cultures. Some variation in receptor localization was noted among the four donor pools examined (Table 1). For example, while EphA2 was observed in the basal layer in 2 out of the 6 gingiva organotypic cultures, it was seen in the basal layer of 4 of the 6 tonsil organotypic cultures. Receptor expression detected by immunoblotting varied between donor pools and biological replicates in agreement with the immunohistochemistry results (S1 Fig). While we did not see CD21 protein expression by immunohistochemistry, we did detect CD21 mRNA by RT-PCR (S2 Fig) PCR. (S2 Fig). Others have reported similar data where CD21 mRNA transcripts were identified in epithelial tissue, but CD21 protein was not detected [42]. We next compared the localization of EBV receptor expression in intact human tonsil to receptor expression in organotypic cultures (Fig 3C). Integrin staining was similar between the organotypic cultures and tonsil tissue. Similar to its expression in the tonsil organotypic cultures, EphA2 was also seen in the basal layer of this tonsil section, suggesting that in tonsil epithelium, EphA2 may not be tightly restricted to the suprabasal layers. There was some MHC II staining in the tonsil epithelium, but MHCII-positive cells did not express the epithelial cell marker cytokeratin 5 (CK5), suggesting they are likely infiltrating immune cells. No CD21 was detected within the epithelium of the tonsil tissue.

**Table 1:** EBV receptor localization in organotypic cultures.

	Donor ^3^Pool	Layer	ITGαV	ITGß1	ITGß6	NRP1	EphA2	MHCII	CD21
**Gingiva**	**1**	^1^ **Suprabasal**	**3/3**	**0/3**	**3/3**	**2/3**	**2/3**	**0/3**	**0/3**
		^2^ **Basal**	**3/3**	**3/3**	**3/3**	**0/3**	**1/3**	**0/3**	**0/3**
	**2**	**Suprabasal**	**3/3**	**3/3**	**3/3**	**1/3**	**2/3**	**0/3**	**0/3**
		**Basal**	**3/3**	**3/3**	**3/3**	**0/3**	**1/3**	**0/3**	**0/3**
**Tonsil**	**3**	**Suprabasal**	**2/3**	**1/3**	**3/3**	**2/3**	**3/3**	**0/3**	**0/3**
		**Basal**	**3/3**	**3/3**	**3/3**	**0/3**	**1/3**	**0/3**	**0/3**
	**4**	**Suprabasal**	**2/3**	**1/3**	**2/3**	**3/3**	**3/3**	**0/3**	**0/3**
		**Basal**	**3/3**	**3/3**	**3/3**	**0/3**	**3/3**	**0/3**	**0/3**

^1^Suprabasal cells were defined as all cells above basal cells that have more elongated cell bodies.

^2^Basal cells were defined as the cell layers with more compact cell bodies.

^3^Two separate donor pools for each cell type were used with three independently generated organotypic cultures from each donor pool. Numbers indicate the number of replicates positive for a given receptor protein.

### EBV can infect stratified epithelium from either the basal or apical surface

In our previous studies, we had scored the organotypic cultures to allow EBV to enter the epithelium, using the rationale that we do not know whether initial infection occurs in cells located near the apical surface or in the basal layers [12]. However, we have not determined whether scoring of the tissue is required for EBV infection. Briefly, scoring entails using a scalpel to slice through the organotypic layers to allow access of the virus to the multiple layers of the organotypic tissue. While our initial efforts were focused on infection from the apical surface, models of the EBV lifecycle propose that EBV, derived from productively replicating B cells, infects the epithelium from the basolateral surface and is ultimately released into the oral cavity [26,28,30,43]. Our model would have greater utility if we could also infect organotypic cultures from the basal surface.

**Fig 4:**
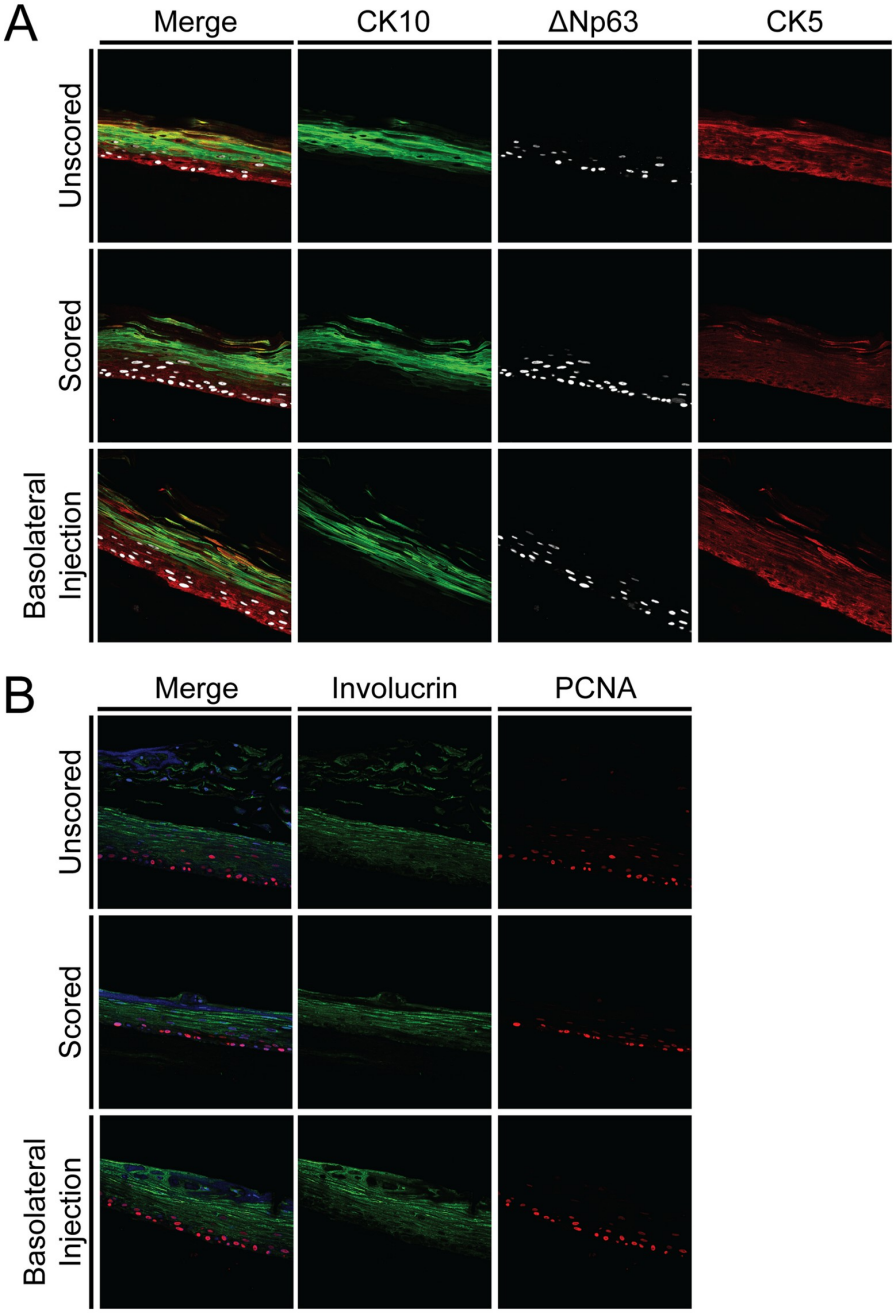
Differentiation of organotypic cultures is not affected by scoring tissue or basolateral infection. Organotypic cultures were collected at 9 days of growth and stained for a variety of differentiation markers. **(A)** Cytokeratin 10 (CK10) was expressed in the upper layers of the epithelium, while ΔNp63 expression was detected in the lower layers of the epithelial model. Cytokeratin 5 (CK5) was expressed in all epithelial cells. **(B)** Involucrin was detected in all but the bottommost layer of the epithelium. PCNA was detected in several of the lower layers of the epithelium.

**Fig 5:**
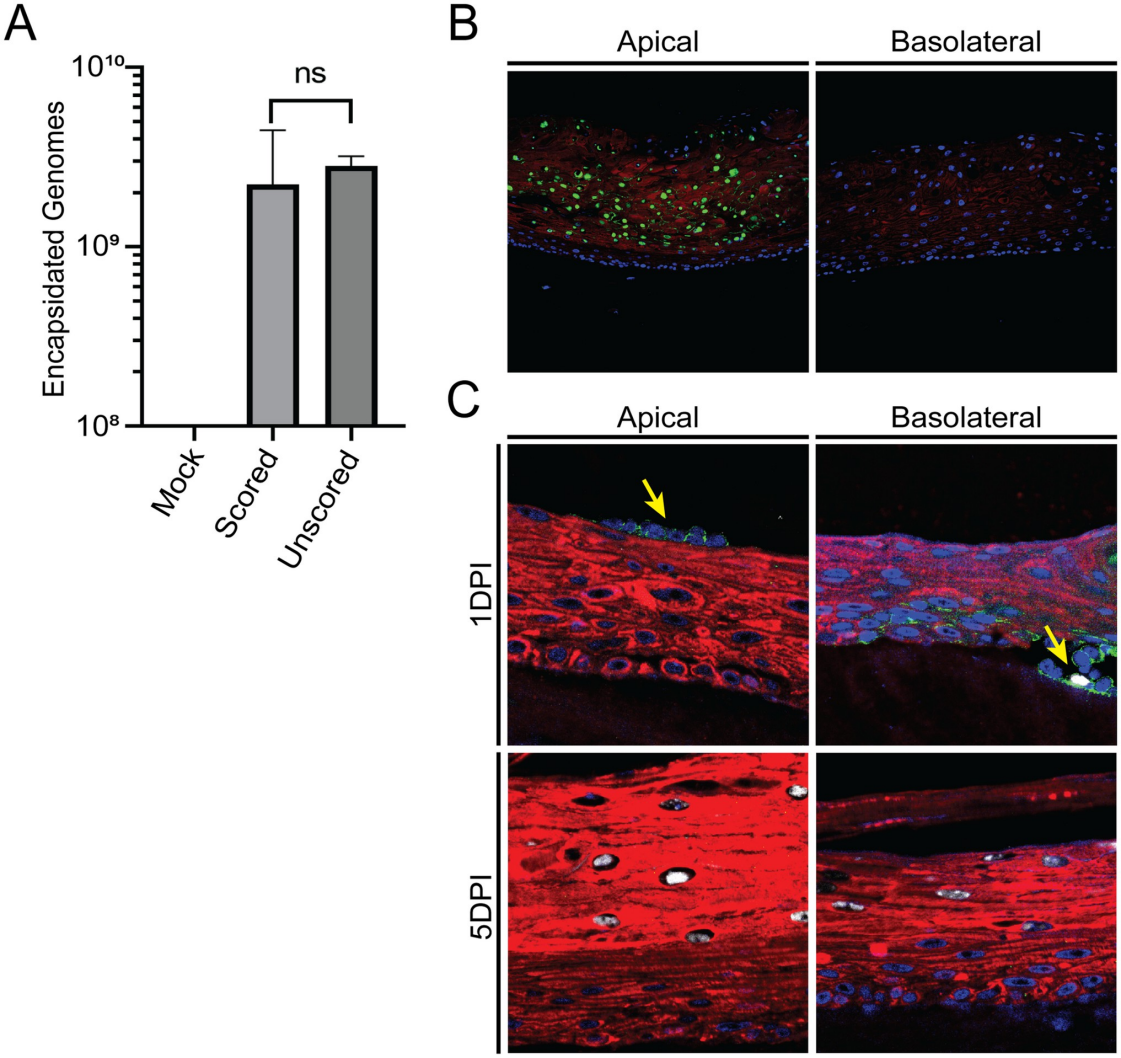
EBV can infect from either the apical or basolateral surface of organotypic cultures without scoring. **(A)** Scored or unscored organotypic cultures were infected with 2.5 x 10^6^ virus-producing Akata cells. Quantification of encapsidated genomes produced by organotypic cultures was determined by PCR 6 DPI. A Welch two sample T-test determined that there was no significant difference (p = 0.418). **(B)** Virus-producing Akata cells were encased in collagen and applied to the apical surface of a 4 day organotypic culture or to the collagen plug prior to addition of keratinocytes. Hoechst (blue) was used to label the nuclei while cytokeratin 5 (red) was used to identify keratinocytes. Viral DNA was detected by *in situ* hybridization (green). **(C)** Virus-producing Akata cells were added to either the apical or basolateral surface of 4 day organotypic cultures. Akata B cells were detected using CD19 staining (green) at 1 DPI (yellow arrows) but were not seen at 5 DPI. Hoechst (blue) was used to label the nuclei while cytokeratin 5 staining (red) identified keratinocytes. Viral DNA was detected by *in situ* hybridization (white).

We first needed to determine whether scoring organotypic cultures or basolateral insertion of virus beneath the stratified tissue would affect differentiation or epithelial cell organization. Organotypic cultures were either unscored, scored, or had warm PBS inserted under the basolateral surface. The stratified tissues were analyzed for expression of the differentiation markers involucrin and cytokeratin 10 (CK10) as well as PCNA or ΔNp63 to identify proliferating undifferentiated basal cells [44–47]. Stratified tissue was formed regardless of whether scoring or basolateral insertion of virus occurred, and all of the cells in the lowest layers of the organotypic cultures were positive for PCNA and ΔNp63 as expected (Fig 4). Furthermore, CK10 had little to no overlap with the undifferentiated cell marker ΔNp63, suggesting CK10 would be an effective marker to identify differentiated epithelial cells in our organotypic cultures. Basolateral infection did seem to result in slightly thinner organotypic cultures (S3 Fig). Normal gingiva *in vivo* does not produce a defined stratum corneum or undergo extensive cornification during stratification [48]. To determine whether scoring of organotypic cultures is essential for EBV infection, the ability of EBV to infect organotypic cultures was examined in unscored or scored organotypic cultures infected by productively replicating B cells added to the apical surface. To quantify infection, qPCR was used to quantify encapsidated EBV genomes in the tissue at 6 days PI (Fig 5A). No significant difference was detected between the infection in scored or unscored organotypic cultures, suggesting that scoring is not necessary.

To model infection from productively replicating B cells localized in the dermis, productively replicating B cells were encased in a thin layer of collagen as a dermal equivalent and placed on top of the collagen plug prior to seeding of epithelial cells. To ensure that the collagen embedding process did not prevent infection, similar collagen-encased B cells were added to the apical surface of 4 day old organotypic cultures as a control. The subsequent presence of EBV DNA within the organotypic cultures indicated that the collagen did not significantly interfere with infection from the apical surface (Fig 5B, left panel). By contrast, no infection was observed when collagen-encased B cells were placed on the collagen plug for basal infection (Fig 5B, right panel). One explanation for the lack of infection via the basolateral surface is that the organotypic cultures had not yet stratified, which may have precluded efficient infection of the keratinocytes. Alternatively, infection may have inhibited cellular proliferation, preventing infected cells from differentiating and allowing the production of virus [14,15,49]. It is also possible that EBV does not efficiently infect the undifferentiated epithelial cells and/or may not have produced sufficient virus to mediate infection.

To examine these possible issues, we determined whether preformed organotypic cultures were susceptible to infection from the basolateral surface by gently separating the edge of the nascent organotypic culture from the collagen plug and inserting a micropipette to introduce non-collagen encased virus-producing B cells directly between the epithelium and collagen. Basolateral infection in this manner was then compared to apical infection with non-encased virus-producing B cells. The presence of B cells was confirmed by staining with CD19 (Fig 5C). Following apical infection, CD19-positive B cells were detected at the apical surface 1 day PI, though no CD19 staining was seen by 5 days PI. Basolateral infection resulted in large pockets of CD19-positive B cells between the epithelium and collagen at 1 day PI, as well as some scattered CD19-positive B cells within the body of the organotypic culture. CD19-positive B cells were not detected at 5 days PI. Using this method to infect at the basal surface resulted in readily detectable infection of the raft culture that was comparable to that observed within the raft infected from the apical surface (Fig 5C, see 5DPI).

### EBV genomes are present within undifferentiated basal cells

**Fig 6:**
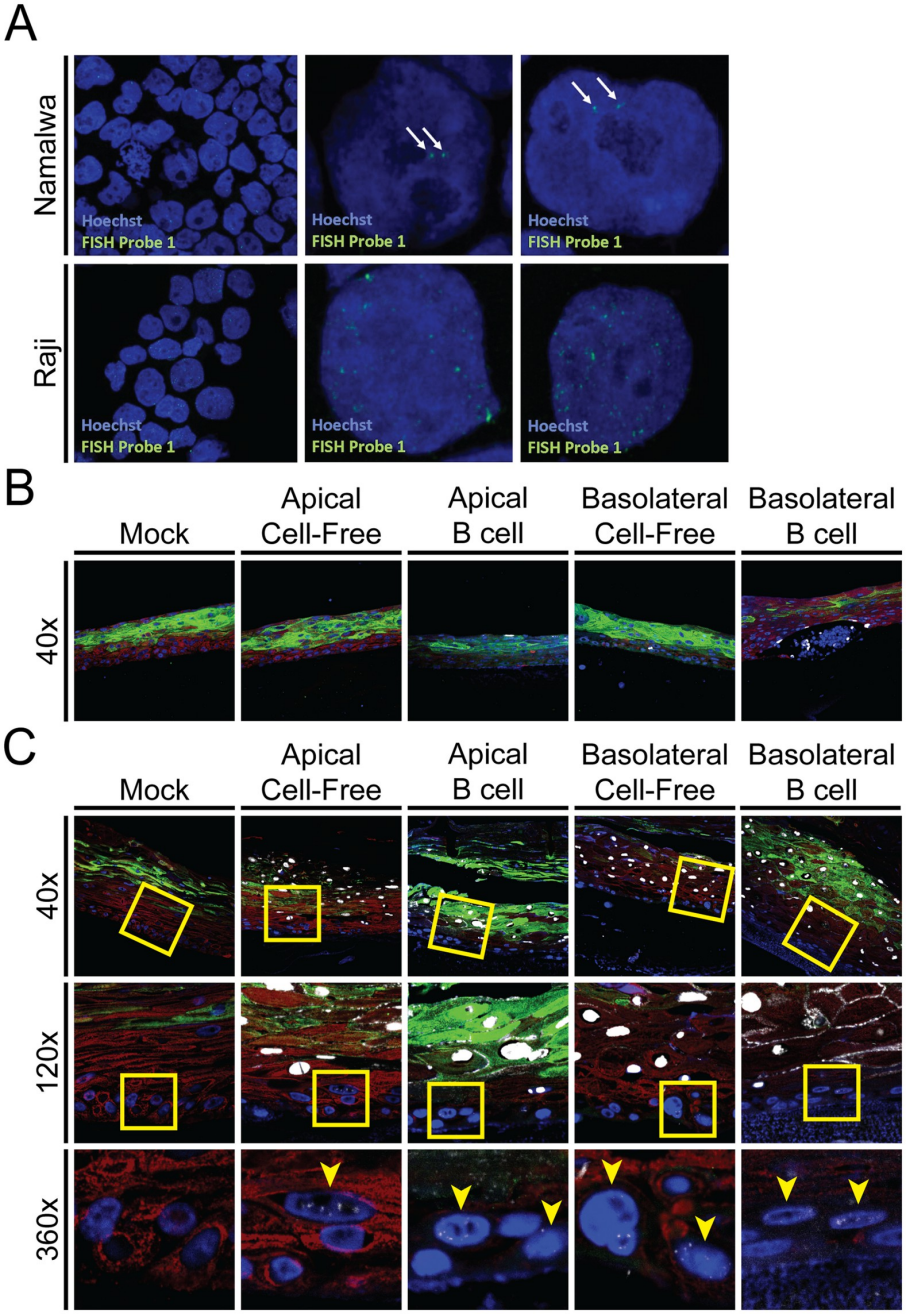
Cell-free and cell-associated EBV can infect cells in both the basal and suprabasal layers when added either to the apical or the basal surface of organotypic cultures. *In situ* hybridization probes targeting the EBV genome were validated to be specific and sensitive by detecting exactly two copies of the EBV genome in Namalwa B cells **(A)**. Gingiva organotypic cultures were infected by B cell-derived cell-free virus or virus-producing B cells at the apical or basolateral surface as indicated. Organotypic cultures were collected at 1 DPI **(B)** and 5 DPI **(C)**. Sections were probed for cytokeratin 10 (green), cytokeratin 5 (red), cell nuclei (Hoechst, blue), and viral DNA (white). EBV was detected by *in situ* hybridization in undifferentiated epithelial cells (CK5 (+) CK10 (-) cells). **(C)** Yellow squares designate areas imaged with enhanced magnification. Yellow arrowheads indicate cells in the lowest layers of the organotypic culture with discrete nuclear puncta. Micrographs are representative of three biological replicates from two distinct donor pools.

Because EBV-encoded noncoding small RNAs or EBERs have been detected in basal epithelial cells in human tissue collected by laser microdissection, we considered the possibility that latent EBV might be present in the basal cells of our organotypic cultures [14]. Although EBV is readily detected in differentiating epithelial cells as a result of productive replication, it is likely that, as in latently infected B cells, only a few copies of the EBV genome would be present within infected basal cells. To determine whether EBV DNA could be detected in the basal layer, gingiva organotypic cultures were infected at 4 days using either B cell-derived cell-free virus or B cell-associated virus at either the apical or basolateral surface. Basal cells were then examined for the presence of EBV genomes by *in situ* hybridization using probes sufficiently sensitive to detect single viral genomes (Fig 6A). Apical infection with cell-free virus did not yield detectable EBV DNA at 1 day PI. There was a limited amount of EBV DNA detected at 1 day PI following basolateral infection with cell-free virus. Apical infection with virus-producing B cells resulted in a small number of EBV DNA-positive signals in CK5-positive epithelial cells in the most apical layer of the epithelium (Fig 6B). By contrast, basolateral infection with virus-producing B cells resulted in more numerous CK5-positive epithelial cells containing EBV DNA (Fig 6B). By 5 days PI, both apical and basolateral infection resulted in readily detectable infection (Fig 6B).

CK10, a marker of epithelial cell differentiation, is found in all differentiated epithelial cells of organotypic cultures (Fig 4A). By one day after basolateral infection with either cell-free or cell-associated virus, EBV DNA could be detected in a few CK5+/CK10- epithelial cells. CK10 staining was inconsistent at this early time point, possibly due to the incomplete differentiation of the organotypic cultures and the relatively low levels of CK10 normally expressed by gingiva [45,46]. By 5 days PI, EBV DNA was detected in epithelial cells in organotypic cultures, regardless of whether infection occurred at the basolateral or apical surface, or whether cell-free virus or virus-producing B cells were used to initiate infection (Fig 6C). CK10 staining was consistently detected in all suprabasal layers at this time point. In CK5+/CK10- cells, EBV DNA was either diffusely localized within the nucleus by *in situ* staining, which may represent cells with active viral genome replication, or as small puncta, which may represent latent or non-productive infection (yellow arrowheads) (Fig 5C). There was robust infection within CK5+/CK10+ epithelial cells, agreeing with our previous findings that EBV readily infects and replicates within differentiated suprabasal cells.

**Fig 7:**
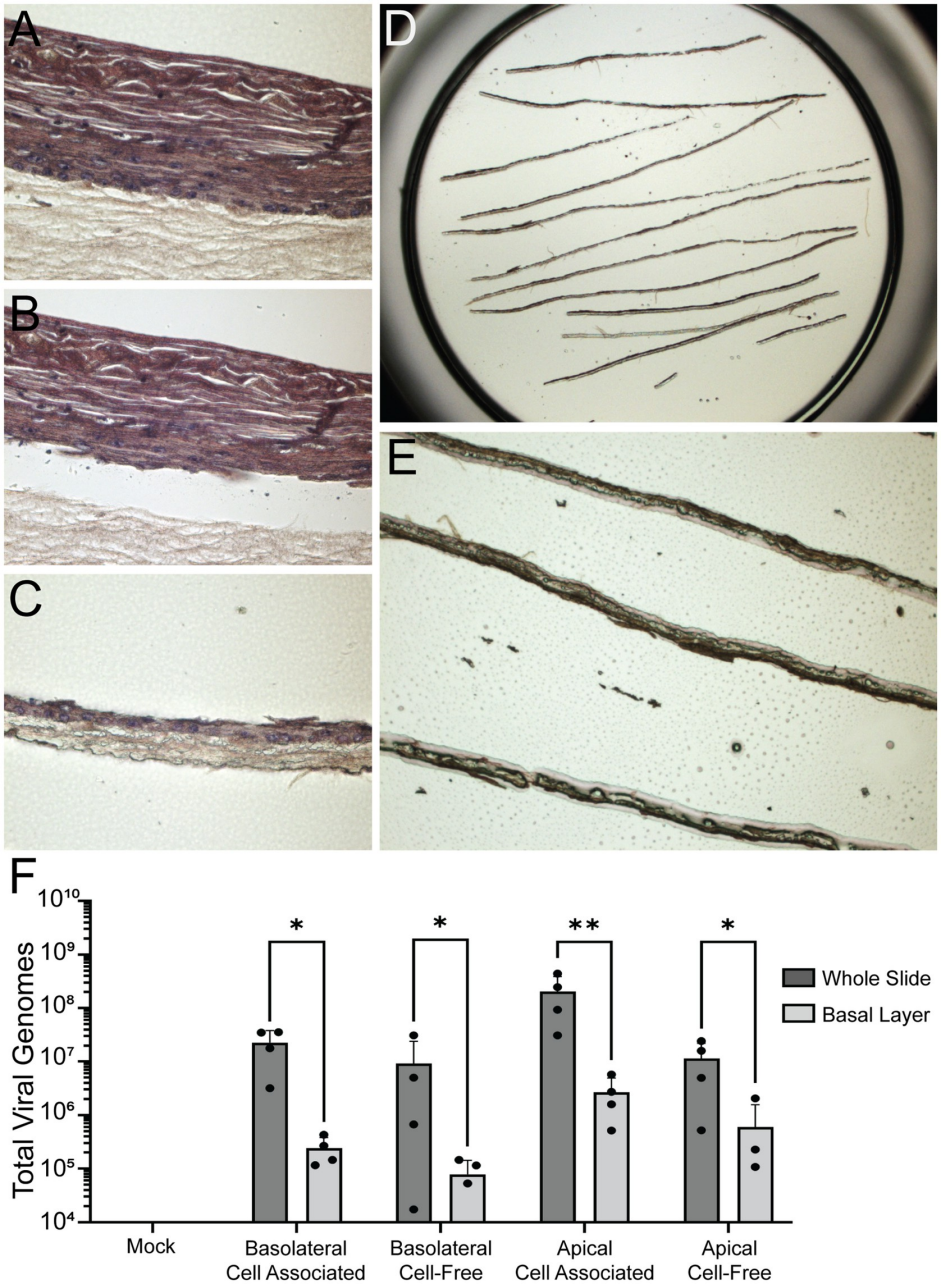
EBV genomes are detected in the basal layer 5 days post infection. Organotypic cultures were infected using either productively replicating B cells or cell-free virus at either the apical or basolateral surface and collected at 5 days post infection. **(A)** Organotypic culture section stained with hematoxylin and eosin viewed prior to capture by LCM. **(B)** View of the organotypic culture section from (A) after capture by LCM. **(C)** View of basal layer captured by LCM. **(D)** View of a single LCM membrane cap containing multiple pieces of captured basal layer from a single organotypic section at 2X magnification. **(E)** 10X magnification of captured basal layer sections in a single LCM cap. **(F)** qPCR quantification of viral genomes present in either an entire organotypic section from a single slide or the captured basal layer from a serial section. There was no statistically significant difference in the ‘whole slide’ or ‘basal layer’ qPCR results from cells infected with productively replicating B cells or cell-free virus (p = 0.315 and p = 0.484, respectively) following analysis by ANOVA. There were statistically significant differences in all infection methods between ‘whole slide’ and ‘basal layer’ (p = 0.013, p = 0.022, p = 0.001, and p = 0.012, respectively) determined by paired t-test. Significance is denoted in (F) by * for p<0.05 and ** for p<0.01.

To validate these findings, sections from organotypic cultures infected by cell-associated or cell-free virus at either the apical or basolateral surface were processed by hematoxylin and eosin staining, and the basal layer was collected by laser capture microdissection (LCM) (Fig 7A–7C). Total DNA was collected from the isolated basal cells (Fig 7C–7E). Total DNA was also collected from an entire adjacent serial section. DNA collected from whole sections or LCM captured basal layers was analyzed by qPCR (Fig 7F). The different methods of infection resulted in similar levels of overall infection (p = 0.315), although there was greater variation in total genomes detected following cell-free virus infection. EBV genomes in the basal layer were approximately two logs lower following infection by any method, indicating that approximately 1% of all viral genomes were within the basal layer.

## Discussion

During the past few years, several laboratories have adopted organotypic cultures to evaluate the EBV lifecycle in stratified epithelium, either using EBV-infected telomerase-immortalized cells or by EBV infection of stratified epithelium using organotypic cultures generated from primary human keratinocytes [12–15]. Using the latter method, we have made several enhancements that result in more authentic representation of EBV infection within epithelium in vivo, thus providing greater utility for the model. While we previously reported that organotypic cultures can model the productive replication of EBV seen in vivo, permissively infected B cells were used to infect the stratified epithelium [12]. This approach was chosen because cell-free EBV does not efficiently infect keratinocytes cultured in monolayers, whereas infection is attained with EBV bound to B cells [24,25]. However, it is possible that in monolayer cells the integrins are not available to be bound by virus. We have determined that B-cell-derived cell-free virus is able to infect organotypic cultures from the basolateral surface, and that both epithelial-derived cell-free and epithelial cell-associated EBV are able to initiate infection when introduced to the apical surface of unscored tissue. These findings allow us to more closely model infection in the human host where both cell-free EBV and isolated epithelial cells are observed in saliva and are thought to infect the apical surface of oral epithelium, while the basal layer is most likely infected in vivo by cell-free EBV released by B cells located beneath the dermis or infiltrating immune cells [26–30]. It was proposed that once gp350 has bound to its cognate receptor on the B cell (CR2), a second EBV glycoprotein is exposed that mediates infection of keratinocytes [23]. Our ability to infect stratified epithelium with cell-free virus suggests that, at least in the context of stratified epithelium, sequestration of gp350 is not required, and that we have generated a biologically relevant model for EBV infection of stratified epithelium that more accurately models infection seen in humans.

We have also validated several observations made in monolayer culture, determining that the proposed epithelial receptors for EBV are expressed in both primary gingiva and tonsil keratinocytes. More importantly, we have shown that these proteins are localized in the same layers of organotypic cultures as in human epithelium, i.e., with integrins primarily present in the basal layers, and NRP1 and EphA2 largely absent in the basal layer but expressed within the suprabasal layers. The localization of different EBV receptors on both the basal and apical surface suggests that EBV can infect from either surface. Based on their localization, it is likely that integrins function as the EBV receptor for infection at the basal layer, whereas it is likely EphA2 and NRP1 function as EBV receptors for infection at the apical surface and within the suprabasal layers where productive replication occurs. The presence of distinct receptors on the apical versus basolateral surface also suggests that different EBV glycoproteins may mediate infection at each surface. The EBV virion contains 11 viral glycoproteins, with gB, gH and gL functioning as the core fusion machinery of the viral particle similar to their homologues present in all herpesviruses [50–52]. Both gH and BMRF2 contain motifs known to mediate binding to integrin proteins [34,53]. Indeed, BMRF2 has been proposed to mediate viral entry via the basal layer through interactions with integrin proteins [21,34]. While some data supports such a role for BMRF2, a virus completely lacking this glycoprotein has not been generated to address this possibility. If other viral protein(s) are found to mediate entry, the cognate receptor, likely EphA2, NRP1, or integrins, can be identified, though it is possible that an as yet unidentified EBV receptor may also contribute to infection. Moving forward, the infection of organotypic cultures with cell-free recombinant EBV in which individual glycoprotein genes have been targeted for deletion or mutation can be used to evaluate the role of the different glycoproteins in infection of stratified epithelium. Using organotypic cultures generated from primary keratinocytes, we found that EBV could infect cells in the basal layer regardless of whether infection was initiated at the basal or apical surface. Similar findings have been reported for the herpes simplex virus and varicella zoster virus [54,55]. This finding suggests the possibility that there may be a broader role for EBV in the epithelium beyond productive replication. One intriguing possibility is that the EBV-infected cells observed in the basal layer might provide a latent reservoir of EBV in the epithelial compartment, which could be either transient or long-lasting [14,38]. To provide a more long-lasting reservoir in basal cells, these cells would likely have to be excluded from the normal pathway for differentiation in stratified epithelium [14,15,49]. The possibility of an epithelial reservoir for EBV distinct from the B cell compartment was suggested by a study of patients treated with Rituximab, which causes a severe depletion of B cells from the circulation [56]. In these patients, shedding of EBV in the oropharynx persisted in the absence of B cells, suggesting the possibility that an epithelial reservoir might exist [56]. The latency-associated EBER transcripts, but not productive genes, are detected in the basal cells of oral hairy leukoplakia biopsies and normal tonsil tissue isolated by laser capture microdissection from formalin-fixed paraffin sections, using reverse transcriptase real-time PCR analysis [14]. While EBV-infected basal cells are also detected in organotypic cultures generated from EBV-infected telomerase-immortalized oral keratinocytes, these cells are selected for the presence of EBV using a selectable marker within the EBV genome prior to formation of the organotypic culture [14].

This model for EBV infection of epithelial cells may also provide better guidance for vaccine development. While there are multiple investigators exploring approaches to EBV vaccines, the majority of these approaches have focused on gp350. Yet it has been demonstrated in monolayer culture that antibodies to gp350 facilitate infection of the epithelium [23]. Even if this does not occur in stratified epithelium, EBV infection of epithelium may not be blocked by targeting gp350, allowing EBV to be amplified by productive replication in the epithelium, likely producing more virus that could then initiate infection of B cells [57]. Recently, EBV has been implicated in the development of multiple sclerosis [58], strengthening the case for generation of an EBV vaccine [58]. The identification of the viral glycoproteins, possibly BMRF2, that mediate EBV infection of epithelium could provide additional targets for vaccination against EBV.

## Materials and methods

### Ethics statement

This study was evaluated by the Pennsylvania State College of Medicine Institutional Review Board and determined not to involve human subjects as defined by federal regulations.

### Cells and cell lines

Akata cells, an EBV-positive Burkitt lymphoma (BL) cell line, were grown in suspension culture in RPMI 1640 medium supplemented with 10% fetal bovine serum. Virus replication was induced in Akata cells by addition of 100 μg/ml anti-human F(ab’)_2_ at 24 or 48 hours prior to infection of organotypic cultures. Mouse J2 3T3 fibroblasts were grown using DMEM supplemented with 10% newborn calf serum and 25 μg/ml gentamicin sulfate.

Lymphocytes were isolated from whole blood by density centrifugation (Lymphocyte Separation Medium; MP Biomedicals, LLC), and B cells were purified using anti-human CD19 microbeads (MACS; Miltenyi Biotec) according to the manufacturer’s instructions. Gingiva and tonsil tissue was obtained from patients undergoing either dental surgery or tonsil removal, respectively. Epithelial tissue from multiple patients was combined and washed with three changes of cold PBS containing 67 μg/ml gentamicin sulfate and 100 units/ml nystatin for 20 to 30 minutes. Connective and dermis tissue were removed with a scalpel and discarded, and the remaining tissue was washed, then minced and dissociated using 0.05% trypsin-EDTA. Trypsin was neutralized with E medium supplemented with 5% FBS [59]. Keratinocytes were cultured to 70% confluence, then harvested and stored in liquid nitrogen for future use. Note that tissue from multiple patients was combined, and thus, the keratinocytes are referred to as donor pools.

### Organotypic culture

Organotypic cultures were generated as previously described [12,60]. For each organotypic culture, 6.25 x 10^5^ J2 fibroblasts were resuspended with 0.25 ml 10x reconstitution buffer and combined with 0.25ml of 10x DMEM, 2 ml of 4.0 mg/ml collagen I (Corning 354236) and 6 μl 10N NaOH, mixed thoroughly by gentle inversion and allowed to harden in a 6-well plate incubated at 37°C. Keratinocytes were expanded in medium K-154 (Gibco 15710–064) supplemented with human keratinocyte growth supplements (Gibco S-001-K) and the Rho kinase (ROCK) inhibitor Y-27632 dihydrochloride (Sigma Y0503). Keratinocytes were dissociated from cell culture plates using 0.05% trypsin-EDTA, washed twice and resuspended in E media containing EGF (Corning 354001) at a concentration of 1 x 10^6^ cells per ml. A total of 2 x 10^6^ keratinocytes were added to the surface of each collagen plug and allowed to reach confluency at 37°C in a 5% CO_2_ atmosphere. To culture the keratinocytes at the air-liquid interface, the collagen plugs were removed from the 6-well plate and placed onto a steel cloth grid in a 10 cm plate, with E medium without EGF applied under the grid. The organotypic culture was then designated as day 0. All organotypic cultures were infected four days later. For cell-associated infection, which was used in most experiments, productively-replicating Akata cells were collected 24 hours post induction then centrifuged, washed and resuspended at a concentration of 2.5 x 10^6^ cells per 200 μl in 37°C PBS. In some experiments where indicated, the apical surface of the organotypic culture was scored multiple times with a sterile scalpel, prior to the addition of productively replicating Akata cells. In other experiments designed to determine whether EBV could infect from the basolateral surface, 2.5 x 10^6^ induced Akata cells were resuspended in 100 μl warm PBS and inserted between the organotypic culture and the collagen plug using a micropipette.

To embed the virus-producing cells in the collagen matrix, Akata cells were resuspended in PBS at a final concentration of 2.5 x 10^6^ cells per 100 μl. The cell suspension was mixed 1:1 with the collagen mixture, with PBS replacing the cell suspension for mock-infected organotypic cultures and added either to the apical surface of a 4 day old organotypic culture or to the top of a previously solidified collagen plug. The samples were immediately incubated at 37°C to allow the collagen to solidify. After 1 hour incubation, keratinocytes were seeded on top of the collagen plugs containing Akata cells.

For cell-free virus infection, medium collected from productively replicating Akata cells was concentrated ~30-fold using 100-kDa cutoff Amicon Ultra-15 spin filters (Millipore UFC910024), and 200 μl was applied to the apical surface of scored organotypic cultures. For basolateral infection with cell-free virus, virus-containing supernatant was concentrated ~60-fold, and 100 μl of concentrated virus was inserted between the epithelial layer and collagen plug using a 200 μl pipet. For experiments without a specified concentration of protected viral genomes, Akata cells carrying wild-type EBV were induced 48 hours prior to infection of organotypic cultures. On the day of infection, supernatant from induced Akata cells was collected, centrifuged at 500 x g for 10 minutes, and passed through a 0.45 μm filter. For experiments with specified amounts of protected viral genomes, viral supernatant was concentrated by tangential flow, and the number of genomes quantified by PCR. High volumes of viral supernatant were concentrated by passing through a MidiKros hollow fiber membrane module with a 500,000 molecular weight cutoff. Concentrated viral stocks were stored at -80°C. Viral titers were calculated using Benzonase-treated supernatants, and DNA was extracted through a standard phenol-chloroform protocol. Concentrated viral stocks were diluted in warm PBS prior to inoculating organotypic cultures.

### Virus extraction from epithelial cells

To generate epithelial-derived virus, infected organotypic cultures were frozen and ground gently with a plastic pestle to form a homogenate, which was clarified by centrifugation at 2500 x g for 10 minutes. The supernatant fraction was collected, and viral titers were determined by PCR. The virus stocks were diluted in PBS and 200–400 μl was added to the apical surface of scored organotypic cultures four days after the organotypic was lifted to the air-liquid interface.

### Infected epithelial cell isolation

To isolate virus producing epithelial cells, scored organotypic cultures were infected with 2.5 x 10^6^ induced Akata cells at 4 days and harvested 6 days later. PBS (1.5 mls) was added to the surface of the organotypic culture and pipetted up and down repeatedly to dissociate epithelial cells loosely attached to the organotypic culture. These cells were pelleted by centrifugation at 2143 x g for 5 minutes, resuspended in 750 μl PBS and passed through a 40 μm nylon cell strainer (BD Falcon 352340). To determine whether organotypic cultures could be infected with virus from epithelial cells, 200 μl of the epithelial cell suspension was added to the apical surface of the 4 day organotypic cultures generated using a different donor pool.

### Cell surface labeling with biotin

Primary tonsil and gingiva epithelial cells were grown to 80% confluence in 60 mm plates. Cells were then washed, incubated with 1 mg/ml EZ-Link Sulfo-NHS-Biotin (Thermo Fisher 21217) at 4°C for 45 minutes, then washed with ice-cold PBS containing 100 mM glycine. Cells were lysed in 500 μl RIPA buffer (150 mM NaCl, 5 mM Tris-HCl pH 8.0, 1% NP40, 0.5% deoxycholate, 0.1% SDS) supplemented with HALT protease inhibitor cocktail (Thermo Fisher 78425). The lysate was incubated with 600 mg of streptavidin-labeled paramagnetic particles (Promega Z5481) for 30 minutes at room temperature. The paramagnetic particles were then collected using a magnetic stand, washed with RIPA buffer supplemented with protease inhibitors, and resuspended in 50 μl of RIPA buffer containing protease inhibitors. The total lysate, unbound, and particle bound fractions were boiled in SDS-PAGE Laemmli buffer and subjected to SDS-PAGE and immunoblot analysis.

### Immunoblotting

Organotypic cultures were placed in 500 μl RIPA buffer containing protease inhibitors, subjected to three freeze-thaw cycles and homogenized using a Dounce homogenizer. The lysate was sonicated on ice at 2.5 W for a total of 30 seconds and clarified by centrifugation at 20,000 x g for 10 minutes at 4°C. Protein concentration was determined using a bicinchoninic acid assay (Thermo Fisher 23225), and gels were loaded with 45μg of total protein per well. Protein was extracted from monolayer cell cultures using RIPA buffer containing protease inhibitors. Lysates were combined with SDS-PAGE Laemmli buffer and boiled for 10 minutes. Proteins were separated on a 7.5% SDS-polyacrylamide gel, transferred to polyvinylidene fluoride membranes (Millipore IPVH00010) using a semidry transfer apparatus (Bio-Rad) at 25V for 60 minutes and semi-dry transfer buffer (50 mM Tris-HCl, 40 mM glycine, 0.04% SDS, 20% methanol). Membranes were blocked using 5% milk in Tris-buffered saline containing 0.1% Tween-20 overnight at 4°C. Antibodies were used at the following dilutions in Tris-buffered saline containing 0.1% Tween-20 supplemented with 0.1% nonfat dry milk and 0.02% sodium azide: cytokeratin 5 (1:20000; Abcam ab52635), EphA2 (1:500; Santa Cruz sc-398832), GAPDH (1:10,000; sc-25778), ITGαV (1:5000; Abcam ab179475), ITGß1 (1:2000; Abcam ab179471), ITGß6 (1:1000; Thermo Fisher PA5-47588), and NRP1 (1:1000; Abcam ab81321). HRP-conjugated secondary antibodies (anti-mouse, Jackson 115-036-062; anti-rabbit, Millipore GENA934; anti-sheep, Millipore AP147P) were used at a dilution of 1:5000 in Tris-buffered saline containing 0.1% Tween-20 and 0.1% milk for 1 hour at room temperature. Imaging was completed using ECL developing reagent (Thermo Fisher 1896327 A/B). Exposure was optimized using ChemiDoc MP (Bio-Rad) and BioRad Image Lab software. Blots were then stripped at 56°C in a rotating hybridization oven.

### Immunocytochemistry

2.5 x 10^5^ cells were seeded into each well of 4-well permanox chamber slides and allowed to reach 80% confluency over 48 hours. Slides were washed in three volumes of warm 37°C Hanks Balanced Salt solution, fixed at room temperature in 4% methanol-free paraformaldehyde for 10 minutes, then washed with and stored in Hanks Balanced Salt solution at 4°C.

Adherent cells were stained with 15 μg/ml wheat germ agglutinin conjugated to tetramethylrhodamine (Thermo Fisher W7024) according to the manufacturer’s instructions prior to permeabilization. Slides were incubated in PBS containing 0.2% Triton X-100 and 10% goat serum for 30 minutes at room temperature, followed by incubation with primary antibodies diluted in PBS containing 0.1% Triton X-100 and 10% goat serum for two hours at room temperature. Primary antibody dilutions were as follow: EBNA2 (1:2, clone PE2 hybridoma supernatant), EphA2 (1:100, Cell Signaling Technologies 6997), ITGαV (1:500, Abcam ab179475), ITGß1 (1:100, Santa Cruz sc-13590), ITGß6 (1:40, Thermo Fisher PA5-47588), and NRP1 (1:100, Abcam ab81321). After washing in PBS containing 0.1% Triton X-100, slides were incubated with secondary antibodies for two hours at room temperature. Nuclei were stained with Hoechst dye (5ng/ml in PBS), and a glycerol-based mounting media containing p-phenylene diamine was applied. Slides were imaged using a Nikon Eclipse Ti scanning laser confocal microscope, and image analysis was performed using the Nikon Elements software suite.

### Immunohistochemistry of paraffin sections

Organotypic tissue was fixed with formalin for 2 hours at room temperature, embedded in paraffin and sectioned into 5-μM thick sections. Formalin-fixed paraffin embedded sections were incubated at 56°C for 30 minutes, deparaffinized in Fisher SafeClear and rehydrated with serial dilutions of ethanol. Antigen retrieval was accomplished by incubation at 95–100°C for 25 minutes in either 10mM sodium citrate, 0.05% Tween-20, pH 6.0 (for ITGαV, ITGß1, and ITGß6) or 1mM EDTA, 0.05% Tween-20, pH 8.0 (for EphA2 and NRP1). Slides were blocked for 30 minutes at room temperature in PBS containing 10% goat serum and 1% Triton X-100, and incubated overnight with primary antibodies at 4°C. Primary antibodies were used at the following dilutions: CD21 (1:5000, Abcam ab75985), cytokeratin 5 (1:1000, Abcam ab52635; 1:20, Thermo Fisher MA5-12596), cytokeratin 10 (1:2500, Abcam ab76318), EphA2 (1:100, Cell Signaling Technologies 6997), ITGαV (1:500, Abcam ab179475), ITGß1 (1:500, Abcam ab179471), ITGß6 (1:40, Thermo Fisher PA5-47588), MHCII (1:100, Santa Cruz, sc-53302) NRP1 (1:100, Abcam ab81321), ΔNp63 (1:100, Abcam ab735), and PCNA (1:25, Santa Cruz sc-7907). Fluorescently-conjugated secondary antibodies (1:200 dilution) were added for two hours at room temperature, followed by incubation with Hoechst (5 ng/ml in PBS) for ten minutes at room temperature. Slides were then processed and imaged as described above.

### In situ hybridization and immunohistochemistry of paraffin sections

To detect EBV DNA, the pDF361 plasmid from the B95-8 EBV genomic library, containing the EBV genomic *Bam*HI-B fragment, was used to generate probes by nick-translation for in situ hybridization. Plasmid DNA was purified from bacterial cultures using the Endo-free Maxiprep kit (Qiagen 12362). Labeling reactions contained 0.05 M Tris-HCl, 5 mM MgCl_2_, 0.05 mg/ml nuclease-free BSA, 20 μl of 0.01 M DTT, 0.04 mM dNTPs, 0.03 mM 594-dUTP (Enzo ENZ-42845), 1 unit RQ1 RNase-free DNase (Promega M6101), 40 units DNA polymerase I (NEB M0209S) and 4 μg plasmid DNA template in a final volume of 200 μL. The reaction solution was split into four equal aliquots and incubated for two hours at 15°C. To stop the reaction, 2 μL of 0.5 M EDTA was added, and DNA was purified using the QuickClean II PCR Extraction Kit (GenScript L00419-50). DNA was eluted in 100 μl DNase- and RNase-free dH_2_O and stored at -20°C. Prior to use, the DNA was precipitated and resuspended in deionized formamide.

The protocol for combining *in situ* hybridization and immunohistochemistry was adapted from a previously described protocol [61]. Formalin-fixed paraffin embedded sections for in situ hybridization were incubated at 56°C for 30 minutes, then deparaffinized. Slides were placed in near boiling (95–100°C) citrate buffer for 25 minutes, allowed to cool at room temperature for 25 minutes, rinsed twice in 2 x SSC buffer (300 mM sodium chloride, 35 mM sodium citrate, pH 7.2), and dehydrated through a series of ethanol washes and then air-dried. Probes generated by nick translation were resuspended in deionized formamide and mixed 1:1 with hybridization solution (600 mM sodium chloride, 70 mM sodium citrate, 200 μg/ml sheared salmon sperm DNA, 40% dextran sulfate). A total of 20 μl of solution containing 100 ng of probe DNA was applied to each slide, incubated at 90°C for 5 minutes, followed by incubation at 37°C overnight. Slides were washed twice for 5 minutes each at 45°C in 2X SCC supplemented with 0.5% Tween-20, followed by two 5 minute washes at 45°C in 0.5X SCC supplemented with 0.5% Tween-20. Slides were then blocked and probed for proteins of interest with primary antibodies as described above with the exception of CK10, which was diluted 1:1000.

### Laser capture microdissection

Organotypic tissue was fixed in formalin and embedded in paraffin as described for immunohistochemistry. Embedded organotypic cultures were sectioned at 5μM thick onto uncharged slides and stained with hematoxylin and eosin, dehydrated and cleared. Stained slides were stored with desiccant to maintain dehydration until use. The basal layer of tissue sections was collected using the infrared laser of an Arcturus XT Laser Capture Microdissection System (Applied Biosciences) and Arcturus CapSure Macro LCM Caps (Thermo LCM0211). Caps were placed in 0.5ml DNase and RNase-free PCR tubes (Thermo N8010737) immediately after collection. DNA was extracted from tissue sections using the PicoPure DNA Extraction Kit (Thermo KIT0103), according to the manufacturer’s instructions. Samples were incubated for 16 hours at 65°C. Whole tissue sections were collected by dewaxing slides in fresh xylene twice for 5 minutes, allowing the slides to air dry, and collecting tissue by scraping the slide with a fresh unused razor blade and placing the tissue in a 0.5ml PCR tube. DNA was extracted from the tissue as described for the basal layer. DNA from the whole tissue or basal layer was analyzed by qPCR as described below.

### Quantitative polymerase chain reaction

DNA was extracted from cells and virus-containing supernatant fractions using the Qiagen Blood and Tissue kit (Qiagen 69504) following the manufacturer’s recommendations. Supernatant was analyzed by diluting 50 μl of raw supernatant with 150 μl of PBS then proceeding to treatment with buffer AL. To isolate DNA from organotypic cultures, tissue was placed in 50 mM sodium phosphate buffer (pH 8.0) and subjected to multiple freeze/thaw cycles and homogenized with a plastic pestle. After adjustment to 2 mM MgCl_2_, the homogenate was digested with 375 units of Benzonase DNA exonuclease (Sigma E1014-25KU) for two hours at 37°C to eliminate non-encapsidated viral DNA. Benzonase was inactivated by adjusting the solution to 1 M NaCl, and the homogenate was clarified by centrifugation. The supernatant fraction was incubated in 10 mM Tris-HCl (pH 7.4), 400 mM NaCl, 10 mM EDTA, 0.1% SDS, and 0.21 mg/ml Proteinase K (Sigma P6556) at 37°C for two hours, followed by phenol-chloroform extraction, ethanol precipitation and resuspension in 100 μl of DNase- and RNase-free dH_2_O. Viral genomes were quantified by real-time polymerase chain reaction amplification of the *BALF5* gene with forward (5’-AGTCCTTCTTGGCTAGTCTGTTGAC-3’) and reverse (5’-CTTTGGCGCGGATCCTC-3’) primers using TaqMan polymerase and a custom *BALF5* probe (5’ FAM CATCAAGAAGCTGCTGGCGGCCT TAMARA 3’) (Applied Biosystems), as previously described. Ten-fold serial dilutions (1 x 10^2^ through 1 x 10^8^) of recombinant EBV Akata BAC (gift of T. Kanda, Aichi Cancer Research Institute, Japan) was used to generate a standard curve.

### RT-PCR

Organotypic cultures were cultured for 4 days after lifting to the air-liquid interface using gingiva donor pools 1 and 2 as well as tonsil donor pool 4. Prior to harvest of the tissue, organotypic cultures were washed with PBS to remove any remaining E-medium. The tissue was separated from the collagen plug and processed using the Qiagen RNeasy Mini Kit (Qiagen 74104) according to the manufacturer’s protocol. The cDNA was generated according to the manufacturer’s protocol, using oligo dT primers in the Invitrogen SuperScript First-Strand Synthesis System for RT-PCR (Invitrogen 11904–018). The cDNA was amplified using CR2 forward (5’-TGATTCTGTGACATTTGCC-3’) and reverse (5’-CCAACAAGCAAGTAACCAG-3’) primers, which generated a 240 base pair product. The PCR product was analyzed on a 0.8% SeaKem GTG agarose gel (Lonza 50070) using GeneRuler 1kb Plus DNA Ladder (Thermo Scientific SM1333) and 6x TnTrack DNA Loading Dye (Thermo Scientific R1161).

### Virus binding and internalization assay

Binding and internalization of virus was assessed as previously described [62]. Briefly, 293T cells and primary keratinocytes were allowed to reach 80% confluence in 60 mm tissue culture plates, chilled at 4°C for one hour, washed with pre-chilled PBS and incubated with 1 ml of chilled virus-containing supernatant for two hours at 4°C. The cells were washed three times with ice cold PBS to remove unbound virus, followed by the addition of warm cell culture media and incubation at 37°C. Mock-infected and time zero samples were collected immediately after washing. Cells were incubated for 45 minutes at 4°C with PBS containing 10 mM EDTA and 1 mg/ml Proteinase K (Sigma P6556) to remove bound virus. Cells were collected by centrifugation at 6000 x g at 4°C, and washed with ice-cold PBS. Total DNA was purified using the Blood and Tissue kit (Qiagen 69504) following the manufacturer’s instructions. DNA was quantified by qPCR targeting the viral *BALF5* gene as described above. *GAPDH* was used to normalize results. Total DNA was collected from known quantities of cells (1 x 10^2^ through 1 x 10^6^) and used to generate a standard curve for *GAPDH*. The total number of *BALF5* genes detected was divided by the total number of cells determined by *GAPDH*.

### Statistical analysis

Fig 1A was analyzed by two-way ANOVA following log transformation of the raw data. Fig 1B was analyzed by two-way ANOVA following square-root transformation of the raw data. Fig 1C was analyzed by ANOVA following log transformation of the raw data. Fig 5A was analyzed by a Welch two sample T-test following a log transformation of the raw data. The data for Fig 7F was processed by log transformation prior to statistical analysis. The difference between methods of infection for either the ‘whole slide’ or ‘basal layer’ qPCR results were analyzed by ANOVA. The differences between ‘whole slide’ and ‘basal layer’ for each infection method was analyzed by paired t-test. Raw data is supplied in Table 1.

## Supporting information

S1 Fig Gingiva and tonsil organotypic cultures express proposed EBV epithelial receptors. Candidate EBV receptors were detected in 4 day organotypic cultures generated from either **(A)** gingiva or **(B)** tonsil. Each lane represents one of three biological replicates from two different donor pools of gingiva or tonsil organotypic cultures, each containing 45 μg of protein. GAPDH was used as a loading control.
(TIF)

S2 Fig CD21 RNA is detected in 4 day organotypic cultures of primary keratinocytes. The CD21 mRNA was analyzed by RT-PCR in RNA extracted from uninfected organotypic cultures collected four days after lifting to the air liquid interface. RNA isolated from Raji cells served as a positive control. Faint CD21 mRNA was detected in gingiva and tonsil organotypic cultures. A no template control (NTC) was used as a negative control. GAPDH was used as a loading control.
(TIF)

S3 Fig Gingiva organotypic cultures are physiologically similar following scoring and basolateral infection. Mock-infected organotypic cultures were collected at 5 days **(A)** or 9 days **(B)**, corresponding to 1 DPI and 5 DPI of infected organotypic cultures. Images are representative of three biological replicates from two different donor pools.
(TIF)

S1 Table Internalization Data for Fig 2. (XLSX)

S2 Table Raw Data for Figs 1–4. (XLSX)
