# Supplementary figures and images for: New insight into Epstein-Barr virus infection using models of stratified epithelium
Source: PLoS Pathog. 2023 Jan 11;19(1):e1011040. doi: 10.1371/journal.ppat.1011040 (PMC9873185; doi:10.1371/journal.ppat.1011040)

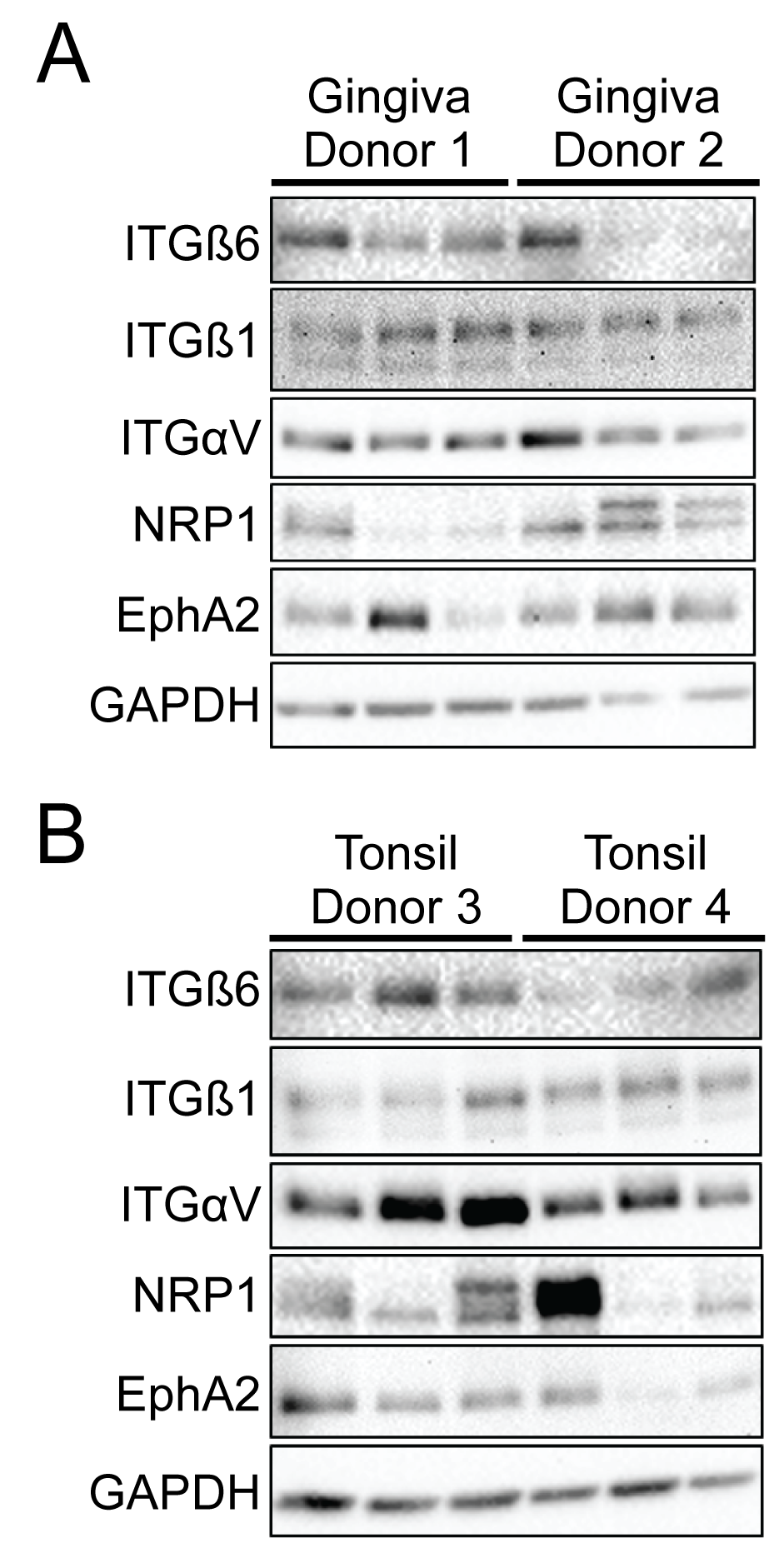

Supplement: S1 Fig — Candidate EBV receptors were detected in 4 day organotypic cultures generated from either (A) gingiva or (B) tonsil. Each lane represents one of three biological replicates from two different donor pools of gingiva or tonsil organotypic cultures, each containing 45 μg of protein. GAPDH was used as a loading control. (TIF) [file ppat.1011040.s001.tif]

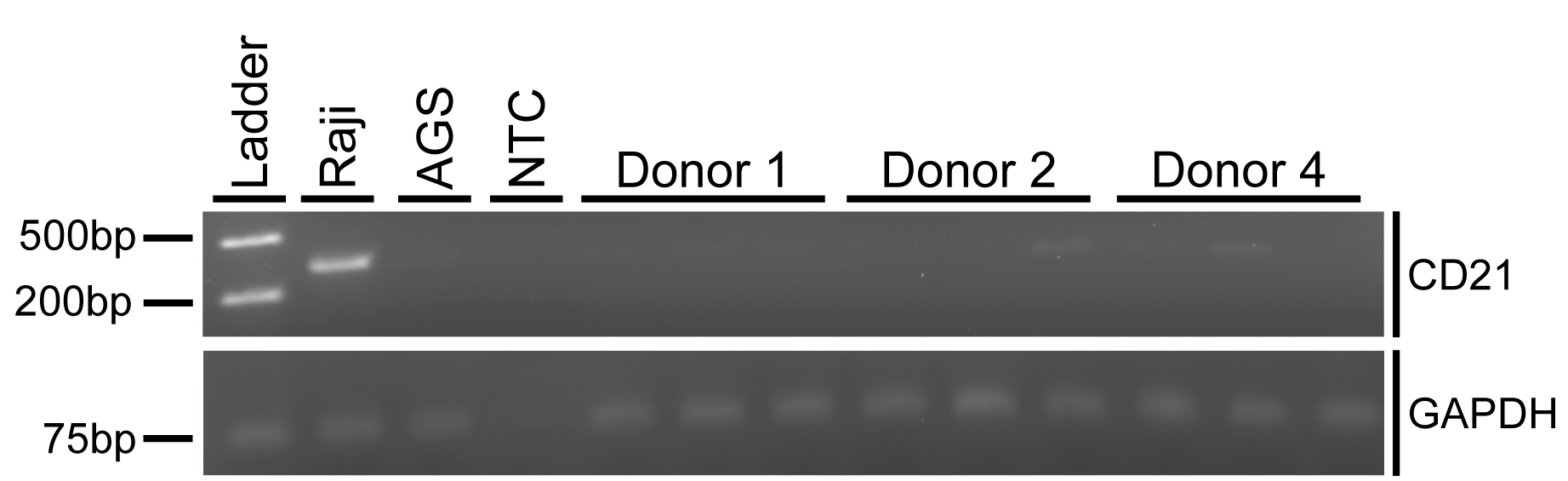

Supplement: S2 Fig — The CD21 mRNA was analyzed by RT-PCR in RNA extracted from uninfected organotypic cultures collected four days after lifting to the air liquid interface. RNA isolated from Raji cells served as a positive control. Faint CD21 mRNA was detected in gingiva and tonsil organotypic cultures. A no template control (NTC) was used as a negative control. GAPDH was used as a loading control. (TIF) [file ppat.1011040.s002.tif]

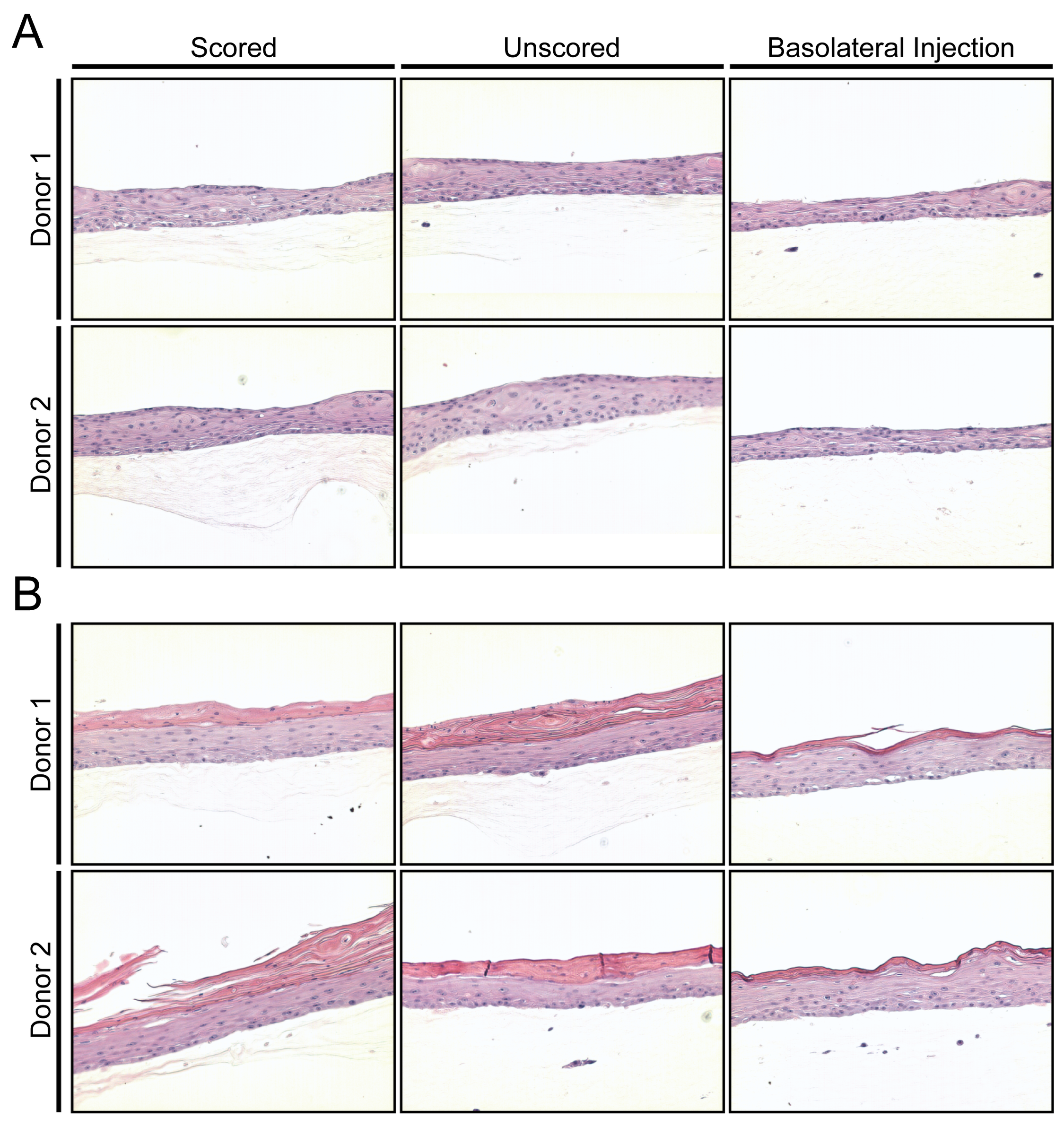

Supplement: S3 Fig — Mock-infected organotypic cultures were collected at 5 days (A) or 9 days (B), corresponding to 1 DPI and 5 DPI of infected organotypic cultures. Images are representative of three biological replicates from two different donor pools. (TIF) [file ppat.1011040.s003.tif]
